# Persistent vs Recurrent Cushing's Disease Diagnosed Four Weeks Postpartum

**DOI:** 10.1155/2022/9236711

**Published:** 2022-08-13

**Authors:** Leena Shah, Emily V. Nosova, Joshua B. Bederson, Khadeen Christi Cheesman

**Affiliations:** ^1^Division of Endocrinology, Diabetes, and Bone Disease, Department of Medicine, Icahn School of Medicine at Mount Sinai, New York, NY, USA; ^2^Department of Neurosurgery, Icahn School of Medicine at Mount Sinai, New York, NY, USA

## Abstract

**Background:**

Cushing's disease (CD) recurrence in pregnancy is thought to be associated with estradiol fluctuations during gestation. CD recurrence in the immediate postpartum period in a patient with a documented dormant disease during pregnancy has never been reported. *Case Report*. A 30-year-old woman with CD had improvement of her symptoms after transsphenoidal resection (TSA) of her pituitary lesion. She conceived unexpectedly 3 months postsurgery and had no symptoms or biochemical evidence of recurrence during pregnancy. After delivering a healthy boy, she developed CD 4 weeks postpartum and underwent a repeat TSA. Despite repeat TSA, she continued to have elevated cortisol levels that were not well controlled with medical management. She eventually had a bilateral adrenalectomy. *Discussion*. CD recurrence may be higher in the peripartum period, but the link between pregnancy and CD recurrence and/or persistence is not well studied. Potential mechanisms of CD recurrence in the postpartum period are discussed below.

**Conclusion:**

We describe the first report of recurrent CD that was quiescent during pregnancy and diagnosed in the immediate postpartum period. Understanding the risk and mechanisms of CD recurrence in pregnancy allows us to counsel these otherwise healthy, reproductive-age women in the context of additional family planning.

## 1. Introduction

Despite a relatively high prevalence of Cushing's syndrome (CS) in women of reproductive age, it is rare for pregnancy to occur in patients with active disease [1]. Hypercortisolism leads to infertility through impairment of the hypothalamic gonadal axis. Additionally, while Cushing's disease (CD) is the leading etiology of CS in nonpregnant adults, it is less common in pregnancy, accounting for only 30–40% of the CS cases in pregnant women [2]. It has been suggested that in CD there is hypersecretion of both cortisol and androgens, impairing fertility to a greater extent, while in CS of an adrenal origin, hypersecretion is almost exclusively of cortisol with minimal androgen production [3]. Regardless of the cause, active CS in pregnancy is associated with a higher maternal and fetal morbidity, hence, prompt diagnosis and treatment are essential.

Pregnancy is considered a physiological state of hypercortisolism, and the peripartum period is a common time for women to develop CD [3, 4]. A recent study reported that 27% of reproductive-age women with CD had onset associated with pregnancy [4]. The high rate of pregnancy-associated CD suggests that the stress of pregnancy and peripartum pituitary corticotroph hyperstimulation may promote or accelerate pituitary tumorigenesis [4–6]. During pregnancy, the circulating levels of corticotropin-releasing hormone (CRH) in the plasma increase exponentially as a result of CRH production by the placenta, decidua, and fetal membranes rather than by the hypothalamus. Unbound circulating placental CRH stimulates pituitary ACTH secretion and causes maternal plasma ACTH levels to rise [4]. A review of the literature reveals many studies of CD onset during the peripartum period, but CD recurrence in the peripartum period has only been reported a handful of times [7–10]. Of these, most cases recurred during pregnancy. CD recurrence in the immediate postpartum period has only been reported once [7]. Below, we report for the first time a case of CD recurrence that occurred 4 weeks postpartum, with a documented dormant disease throughout pregnancy.

## 2. Case Presentation

**Figure 1:**
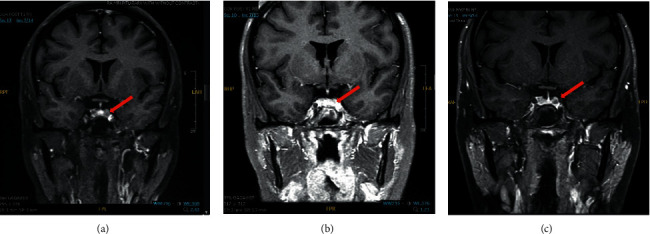
(a) Initial: MRI pituitary with and without contrast showing a coronal T1 postcontrast image immediately prior to our patient's pituitary surgery. The red arrow points to a 3 × 3 × 5 mm hypoenhancing focus representing a pituitary microadenoma. (b) Postsurgical: MRI pituitary with and without contrast showing a coronal T1 postcontrast image obtained three months after transsphenoidal pituitary surgery. The red arrow shows that a hypoenhancing focus is no longer seen and has been resected. (c) Postpartum: MRI pituitary with and without contrast showing a coronal T1 postcontrast image obtained four weeks postpartum. The red arrow points to a 3 mm relatively hypoenhancing lesion representing a recurrent pituitary adenoma.

A 30-year-old woman initially presented with prediabetes, weight gain, dorsal hump, abdominal striae, depression, lower extremity weakness, and oligomenorrhea with a recent miscarriage 10 months ago. Diagnostic tests were consistent with CD. Results included the following: three elevated midnight salivary cortisols: 0.33, 1.38, and 1.10 *μ*g/dL (<0.010–0.090); 1 mg dexamethasone suppression test (DST) with cortisol 14 *μ*g/dL (<1.8); elevated 24 hr urine cortisol (UFC) measuring 825 *μ*g/24 hr (6–42); ACTH 35 pg/mL (7.2–63.3). MRI of the pituitary gland revealed a left 4 mm focal lesion (Figure 1(a)). After transsphenoidal resection (TSA), day 1, 2, and 3 morning cortisol values were 18, 5, and 2 *μ*g/dL, respectively. Pathology did not show a definitive pituitary neoplasm. She was rapidly titrated off hydrocortisone (HC) by six weeks postresection. Her symptoms steadily improved, including improved energy levels, improved mood, and resolution of striae. She resumed normal menses and conceived unexpectedly around 3 months post-TSA. Hormonal evaluation completed a few weeks prior to her pregnancy indicated no recurrence: morning ACTH level, 27.8 pg/mL; UFC, 5 *μ*g/24 hr; midnight salivary cortisol, 0.085 and 0.014 *μ*g/dL. Her postop MRI at that time did not show a definitive adenoma (Figure 1(b)). During pregnancy, she had a normal oral glucose tolerance test at 20 weeks and no other sequela of CD. Every 8 weeks, she had 24-hour urine cortisol measurements. Of these, the highest was 93 *μ*g/24 hr at 17 weeks and none were in the range of CD (Table 1). Towards the end of her 2^nd^ trimester, she started to complain of severe fatigue. Given her low 24 hr urine cortisol level of 15 *μ*g/24 hr at 36 weeks gestation, she was started on HC. She underwent a cesarean section at 40 weeks gestation for oligohydramnios and she subsequently delivered a healthy baby boy weighing 7.6 pounds with APGAR scores at 1 and 5 minutes being 9 and 9. HC was discontinued immediately after delivery. Around four weeks postpartum she developed symptoms suggestive for CD. Diagnostic tests showed an elevated midnight salivary cortisol of 0.206 and 0.723 *μ*g/dL, and 24-hour urine cortisol of 400 *μ*g/24 hr. MRI pituitary illustrated a 3 mm adenoma in the left posterior region of the gland, which was thought to represent a recurrent tumor (Figure 1(c)). A discrete lesion was found and resected during repeat TSA. Pathology confirmed corticotroph adenoma with MIB-1 < 3%. On postoperative days 1, 2, and 3, the cortisol levels were 26, 10, and 2.8 *μ*g/dL, respectively. She was tapered off HC within one month. Her symptoms improved only slightly and she continued to report weight gain, muscle weakness, and fatigue. Three months after repeat TSA, biochemical data showed 1 out of 2 midnight salivary cortisols elevated at 0.124 *μ*g/dL and elevated urine cortisol of 76 *μ*g/24 hr. MRI pituitary demonstrated a 3 × 5 mm left enhancement, concerning for residual or enlarged persistent tumor. Subsequent lab work continued to show a biochemical excess of cortisol, and the patient was started on metyrapone but reported no significant improvement of her symptoms and only mild improvement of excess cortisol. After a multidisciplinary discussion, the patient made the decision to pursue bilateral adrenalectomy, as she refused further medical management and opted against radiation given the risk of hypogonadism.

## 3. Discussion

The symptoms and signs of Cushing's syndrome overlap with those seen in normal pregnancy, making diagnosis of Cushing's disease during pregnancy challenging [1]. Potential mechanisms of gestational hypercortisolemia include increased systemic cortisol resistance during pregnancy, decreased sensitivity of plasma ACTH to negative feedback causing an altered pituitary ACTH setpoint, and noncircadian secretion of placental CRH during pregnancy causing stimulation of the maternal HPA axis [5]. Consequently, both urinary excretion of cortisol and late-night salivary cortisol undergo a gradual increase during normal pregnancy, beginning at the 11^th^ week of gestation [2]. Cushing's disease is suggested by 24-hour urinary-free cortisol levels greater than 3-fold of the upper limit of normal [2]. It has also been suggested that nocturnal salivary cortisol be used to diagnose Cushing's disease by using the following specific trimester thresholds: first trimester, 0.25 *μ*g/dL; second trimester, 0.26 *μ*g/dL; third trimester 0.33, *μ*g/dL [11]. By these criteria, our patient had no signs or biochemical evidence of CD during pregnancy but developed CD 4 weeks postpartum.

A recent study by Tang et al. proposed that there may be a higher risk of developing CD in the peripartum period, but did not test for CD during pregnancy, and therefore was not able to definitively say exactly when CD onset occurred in relation to pregnancy [4]. Previous literature suggests that there may be a higher risk of ACTH-secreting pituitary adenomas following pregnancy as there is a significant surge of ACTH and cortisol hormones at the time of labor. This increased stimulation of the pituitary corticotrophs in the immediate postpartum period may promote tumorigenesis [6]. It has also been suggested that the hormonal milieu during pregnancy may cause accelerated growth of otherwise dormant or small slow-growing pituitary corticotroph adenomas [4, 5]. However, the underlying mechanisms of CD development in the postpartum period have yet to be clarified. We highlight the need for more research to investigate not only the development, but also the risk of CD recurrence in the postpartum period. Such research would be helpful for family planning.

## 4. Conclusion

Hypothalamic-pituitary-adrenal axis activation during pregnancy and the immediate postpartum period may result in higher rates of CD recurrence in the postpartum period, as seen in our patient. In general, more testing for CS in all reproductive-age females with symptoms suggesting CS, especially during and after childbirth, is necessary. Such testing can also help us determine when CD occurred in relation to pregnancy, so that we can further understand the link between pregnancy and CD occurrence, recurrence, and/or persistence. Learning about the potential mechanisms of CD development and recurrence in pregnancy will help us to counsel these reproductive-age women who desire pregnancy.

## Figures and Tables

**Table 1:** 24-hour urine-free cortisol measurements collected approximately every 8 weeks throughout our patient's pregnancy.

Time in relation to pregnancy	4 months before conception and 2 months pre-TSA	2 weeks before conception and 7 weeks post-TSA	10 weeks	17 weeks	29 weeks	36 weeks	4 weeks postpartum
24-hour urine-free cortisol (*μ*g/24 hr) nonpregnant reference range (LabCorp): 6–42 *μ*g/24 hr	825.6	5	34	93	29	15	408

^*∗*^Pregnancy reference ranges have not been well established. UFC levels are typically higher in pregnancy, up to twice the upper limit of normal. Our patient was therefore said to have low UFC levels at 36 weeks gestation.

## Data Availability

The data used to support the findings of this study are included within the article.

## Abbreviations

CD: Cushing's disease
TSA: Transsphenoidal resection
DST: Dexamethasone suppression test
ACTH: Adrenocorticotropic hormone
MRI: Magnetic-resonance imaging
HC: Hydrocortisone
CTH: Corticotroph-releasing hormone
HPA: Hypothalamic-pituitary-adrenal.

## Additional Points

*Note*. Peripartum refers to the period immediately before, during, or after pregnancy and postpartum refers to any period after pregnancy up until 1 year postdelivery.
